# Objective Falls Risk Assessment Using Markerless Motion Capture and Representational Machine Learning

**DOI:** 10.3390/s24144593

**Published:** 2024-07-16

**Authors:** Sean Maudsley-Barton, Moi Hoon Yap

**Affiliations:** Department of Computing and Mathematics, Manchester Metropolitan University, Manchester M15 6BH, UK; s.maudsley-barton@mmu.ac.uk

**Keywords:** anomaly detection, computer-aided diagnosis, falls risk, LSTM autoencoder, representational model, markerless motion capture

## Abstract

Falls are a major issue for those over the age of 65 years worldwide. Objective assessment of fall risk is rare in clinical practice. The most common methods of assessment are time-consuming observational tests (clinical tests). Computer-aided diagnosis could be a great help. A popular clinical test for fall risk is the five times sit-to-stand. The time taken to complete the test is the most commonly used metric to identify the most at-risk patients. However, tracking the movement of skeletal joints can provide much richer insights. We use markerless motion capture, allied with a representational model, to identify those at risk of falls. Our method uses an LSTM autoencoder to derive a distance measure. Using this measure, we introduce a new scoring system, allowing individuals with differing falls risks to be placed on a continuous scale. Evaluating our method on the KINECAL dataset, we achieved an accuracy of 0.84 in identifying those at elevated falls risk. In addition to identifying potential fallers, our method could find applications in rehabilitation. This aligns with the goals of the KINECAL Dataset. KINECAL contains the recordings of 90 individuals undertaking 11 movements used in clinical assessments. KINECAL is labelled to disambiguate age-related decline and falls risk.

## 1. Introduction

Falls are common for all ages, but for older people, without any associated conditions (e.g., Parkinson’s, Multiple sclerosis), the effect of ageing on the motor control system can mean coordination patterns change with age. In turn, this can lead to a propensity to lose balance, resulting in a fall. Deaths due to complications after a fall are the most common cause of death for those aged over 65 [1,2]. Around a third of people aged 65 and over, and over half of those aged over 80, fall at least once a year [3,4]. In addition to the initial injuries, complications from a fall can have a devastating effect on the quality of life of individuals [4]. Several studies have shown that functional movement can be improved with appropriate intervention, such as balance and strength training, leading to fewer future falls [5,6,7,8]. In the UK, falls clinics have been set up to provide this type of care. However, there is no systematic screening for potential fallers. The use of computer-aided diagnosis could make this a possibility, enabling prophylactic intervention. Currently, the National Institute for Health and Care Excellence (NICE) [9] recommend that the way for healthcare professionals to assess falls risk is to routinely ask older people in their care how many times they have fallen in the last 12 months, along with the falls’ context and characteristics. Interestingly, in their 2019 review of these guidelines [10], NICE suggested that new technologies could enhance fall risk assessment. This study proposes a method which could address this need.

If someone is found to have a history of recurrent falls, the most common method of assessing the physical factors that contribute to falls risk is to carry out a clinical test. Common tests include the short physical performance battery (SPPB) [11], timed up and go (TUG) [12], 3 m walk [13] and five times sit to stand (STS-5) [14]. All of these tests have been linked to the assessment of falls risk [14,15,16,17,18,19,20]. These tests are carried out by trained staff, which presents issues for both throughput and variance between staff [21].

Previously, efforts have been made to automate clinical tests. Despite its seeming simplicity, the sit-to-stand movement requires the coordination of many elements of the musculoskeletal system, which is essential for activities of daily living. Ward et al. [14] undertook a 4-year longitudinal study (*n* = 755, mean age of 78.1 ± 5.4) that looked at SPPB as a predictor of injurious falls. They concluded that the STS-5 alone was all that was required to assess falls risk. They suggested that a time taken to complete the test of ≥16.7 s for the STS-5 test may be sufficient to identify those at risk of future falls. Tiedemann et al. [20] also found that the time taken to complete the STS-5 test was an excellent predictor of falls. They found that even a single sit-to-stand could provide a useful indication of falls-risk. Ejupi et al. [19] used a Kinect camera to record community-dwelling older adults carrying out the STS-5 test. They found that sit-to-stand velocity was a better discriminator of falls risk than the time taken to complete the test. In addition, the STS-5 test is particularly well suited to situations with limited space, such as doctors’ offices. Hence, we have chosen to use this movement in this study.

The studies listed above used time or the derivative of displacement with time to assess fallers. However, this approach provides only a rough test for impairment. More informative data can be obtained by considering the coordination of the joints needed to achieve this movement. Markerless motion capture provides a means of collecting this type of information away from the lab, and it is the method we use here. In addition, we also utilised a deep neural autoencoder, trained in an unsupervised fashion on healthy adults from the KINECAL dataset. This allowed the model to learn a compact representation of what a healthy sit-to-stand looks like. From this model, we were able to develop a unique scale that we used to identify participants labelled as at risk in the KINECAL dataset. KINECAL contains the recordings of 90 individuals undertaking movements used in clinical assessments. KINECAL is labelled to differentiate between age-related decline and falls risk. More details of the labelling scheme are shown in the *Data Source* section.

This paper presents the following contributions:

- A method for objective assessment for falls risk, based on a representational model;
- A novel scale of falls risk, which can allow for computer-aided diagnosis of those at risk of falls and track their progress after an intervention;
- This is in contrast to classification models, which reduce the choice to a binary choice of faller or non-faller;
- We demonstrate the use of this method to identify those at risk of falls from the KINECAL dataset [22].

This paper is structured as follows: Section 2 looks at related work, highlighting the motivation for our research. Section 3 details the methodology. Section 4 presents the results. Section 5 provides a discussion of the study. Finally, Section 6 concludes our research, highlighting limitations and areas for future work.

## 2. Related Work

The use of machine models for falls risk assessment tends to focus on classification models [23,24]. However, this approach insists on a hard cut-off: non-fallers vs. fallers. This may not be the most informative approach, especially the use case of the rehabilitation of those identified as at risk of falls. Therefore, a more useful approach is to use a distance metric relative to a healthy norm. This type of scoring can better aid clinicians in tracking an individual’s falls risk over time. Hence, this is the approach used in the current study.

Outside of falls risk assessment, the use of a distance measure, which relates to an ideal form, has been used by other studies. Anton et al. [25] used a distance measure derived from dynamic time warping to assess if a particular exercise had been carried out to the required standard during rehabilitation. In a similar way, Gholami et al. [26] used dynamic time warping in the creation of a distance measure to quantify the degree of dissimilarity between the gait cycle of patients with Multiple Sclerosis and healthy patients of a similar age.

Houmanfar et al. [27] demonstrated that a model-based approach outperformed distance metrics calculated from manually selected features. They used a Hidden Markov Model. Capecci et al. [28] also demonstrated that a Hidden Semi-Markov Model outperformed dynamic time warping for monitoring rehabilitation. In both these studies, the author used a distance metric to compare normal populations to the affected population and proposed that this approach proved more useful than a classification model.

Inspired by this work, we also propose the use of a distance measure derived from a machine model—in our case, a deep autoencoder. An autoencoder was selected because while Markov-chain-like models can only model linear relationships, autoencoders can model non-linear ones. The autoencoder was built using long short-term memory (LSTM) units. This selection was motivated by the time series nature of the data.

Initially, we used Euclidean distance, derived from reconstruction error, as a distance measure. However, this metric alone did not prove adequate to separate those at risk of falls from those not at risk of falls. Hence, we developed a unique scoring system which uses the variance between repetitions as a key indicator of fall risk.

The rest of this paper is devoted to demonstrating the use of this method.

## 3. Materials and Methods

### 3.1. Data Source

The data used in this study are derived from the KINECAL dataset [22]. In particular, we used the data from the five times sit-to-stand STS-5 test.

KINECAL splits its data into several classes: **Healthy Adults** (<65 years, no history of falls in the last 12 months), **Non-Faller** (≥65 years, no history of falls in the last 12 months), **Self-reported Faller_s** (≥65 years, reported 1 fall, in the last 12 months), **Self-reported-Faller_m** (≥65 years, reported >1 fall, in the last 12 months), **Clinically At-Risk** (≥65 years identified as impaired by ≥2 clinical tests (see [22] for detail of the clinical tests used)).

Labelling the data in this way allows for the identification of various sub-sets. Such as those over 65 who have reported multiple falls. Or those over 65 who have not reported a fall. This can be useful when trying to ascertain age-related changes vs. impairment-related changes.

### 3.2. Calculation of Joint Angles

**Figure 1:**
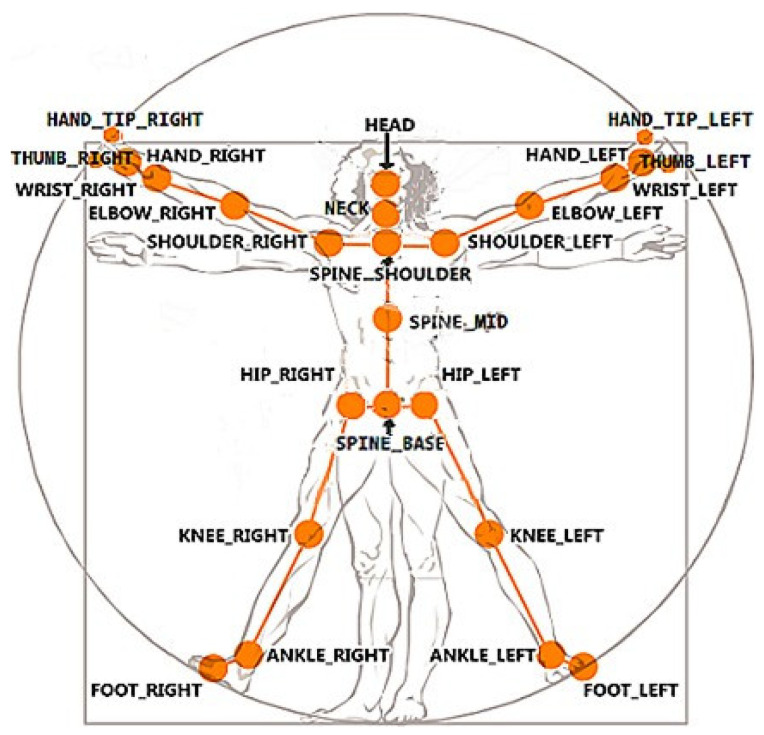
**Kinect joints:** Illustration of the full list of the Kinect joints. Reproduced from Microsoft.

**Figure 2:**
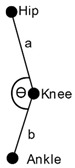
**Knee Angle:** Illustration of how the knee joint angle was calculated in the sagittal plane. The angles in the other two planes are calculated in a similar way.

KINECAL provides 25 three-dimensional joint locations for each frame (Figure 1 shows a full list). From these data, two-dimensional angles were calculated for each of the anatomical planes (sagittal, frontal and transverse). This provided three values for each joint at each time point. Equation (1) was used to calculate the angle between two vectors (*a* and *b*), shown in Figure 2. This represents the angle as the two segments of a limb meeting at a joint.

(1) $$\Theta =\arccos=(\frac{a\times b}{\left||a||\;||b|\right|})$$

### 3.3. Segmentation of Repetitions

**Figure 3:**
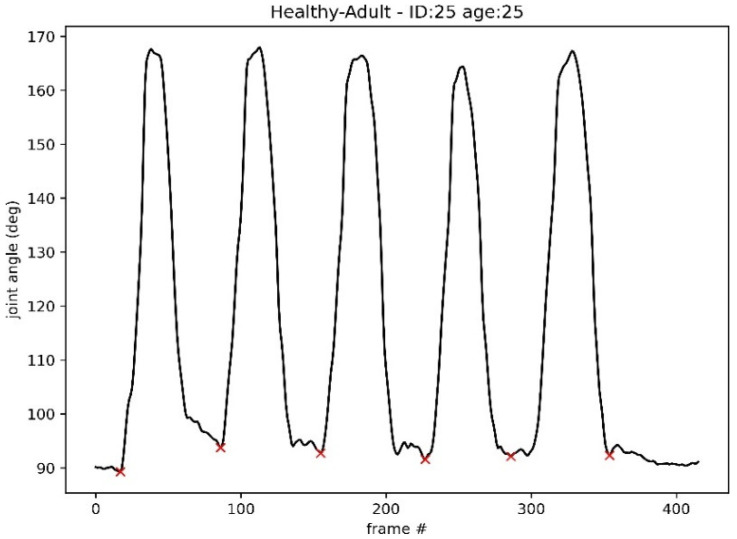
**A graph of right knee angle:** The graph demonstrates how the right knee angle, in the sagittal plane, of a healthy 25-year-old participant changes over time while carrying out the STS-5 movement. A red × identifies the start and end of each repetition. These points were identified using the *argrelextrema* method of the scipy signal Python library. The frame number is denoted by frame # in the figure.

Each recording of the STS-5 test contains 5 repetitions. Figure 3 shows how the right knee angle in the sagittal plane (marked up as KNEERIGHT_a_SP in the Kinecal dataset) changes over the duration of the movement. For each participant, this channel was used in a multi-step segmentation process to extract single repetitions.

The process was as follows: (1) **identify all the valleys in the recording:** the valleys in the recording were identified using *argrelextrema* method of the scipy signal Python library [29]; (2) **snip a singlerep:** a single repetition was identified as the area between the start and end of a valley (indicated by a red ×, in Figure 3); (3) **validate the snipped repeat:** the start and end angle of the snipped repetitions were compared, and if the angle differed by more than 30 degrees, that repetition was rejected (this captured odd start and end chunks when the recording captured artefacts that are unrelated to the sit-to-stand movement); and (4) **visually inspect the single snipped repetitions:** a final visual inspection of each repetition was carried out and any oddities were removed. Resampling and padding.

The time to complete one repetition of the STS-5 movement varied among individuals. Hence, so did the number of recorded frames per repetition (ranging from 54 to 150 frames). The average for a member of the **Healthy Adult** class was 80 frames. To provide a standard basis for comparison, each repetition was resampled to 80 frames. The resampling was achieved by Fourier Transform resampling [30], implemented via the *signal.resample* method of the scipy python library [29]. This process also centred the movement.

**Figure 4:**
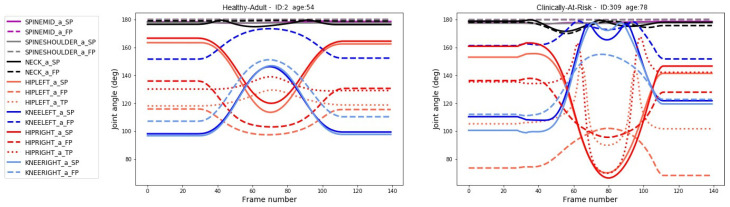
**Joint angle for a single rep:** Illustration of the changes in joint angles over a single repetition for **Healthy Adult** (**left**) and **Clinically At-Risk** (**right**). *Note, the first 30 and last 30 frames are clipped before the distance is calculated. These early and late frames were added as part of the padding process and do not represent the movement*.

Resampled repetitions were padded with a 2 s buffer (60 frames) on either side of the main movement; two example outputs of this process are shown in Figure 4. The padding helped the autoencoder to learn by providing a lead-in before the movement.

The result was a set of 200 frames by 75 channels (25 joints × 3 planes) time series. To aid training, the samples were normalised using the *normalization* function from scikit-learn [31].

Not all of the 75 channels were used in the final model. The most informative channels were selected using cross-validation. The 16 most informative channels represented the following joints and planes: SPINE_MID, sagittal and frontal; SPINE_SHOULDER, sagittal and frontal; NECK sagittal and frontal; HIP_LEFT sagittal, frontal and trans- verse; HIP_RIGHT sagittal, frontal and transverse; KNEE_LEFT, sagittal and frontal; KNEE_RIGHT, sagittal and frontal.

### 3.4. Autoencoder

**Figure 5:**
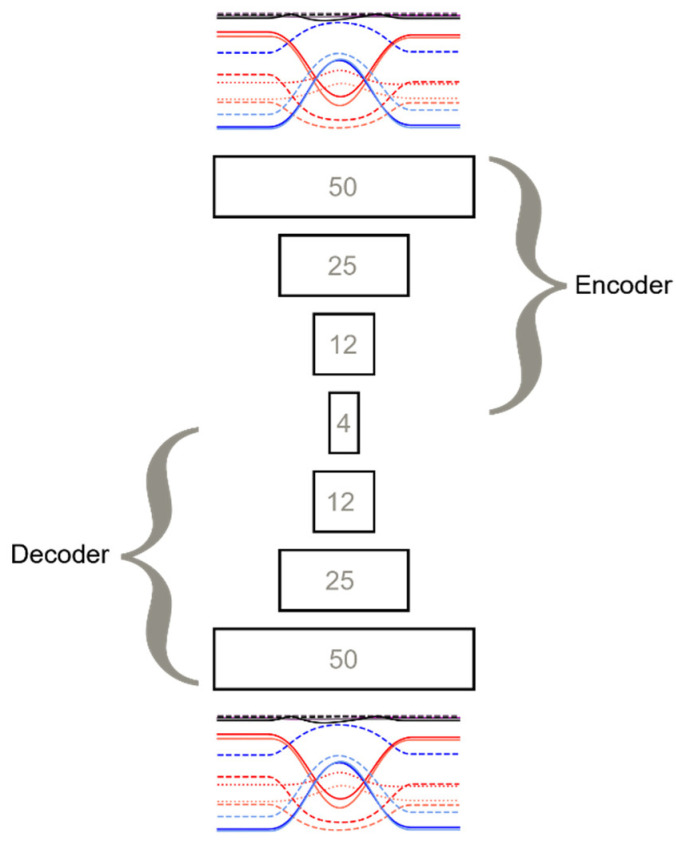
**Autoencoder:** The autoencoder consists of a 3-layer encoder and a 3-layer decoder. The latent representation is 4 LSTM units. The input time series was 200 frames by 16 channels; these were also the dimensions of the reconstructed output. It was built using TensorFlow.

Autoencoders are a class of neural networks which are trained using an unsupervised approach. Autoencoders consist of two parts: an encoder and a decoder. The purpose of the encoder is to compress the input to a latent representation. The decoder then reconstructs the original input from the latent representation. This may sound like a trivial task. However, due to the “bow tie” structure of an autoencoder (Figure 5), the network is forced to encode the time series into fewer and fewer neurons in the encoding section and then tries to recreate the original signal in the decoder section. During the training process, an autoencoder builds a generalised internal representation of the training data, i.e., a representational model.

The number of layers and the number of LSTM units in each layer were selected using the *GridSearchCV* method of scikit-learn [31]. The structure of the final autoencoder is shown in Figure 5. The numbers shown in each layer refer to LSTM units.

### 3.5. Training

The autoencoder was trained to accurately recreate the movements (200 frames by 16 channel time series, defined above) of the **Healthy Adults** group; the loss metric was the mean square error. The mean square error loss of the validation set was used as a signal of an early stopping method, which stopped training if the validation loss remained constant or rose for 50 consecutive epochs.

The trained model could reconstruct unseen movements from the **Healthy Adult** group with a low error rate. However, if asked to reconstruct unseen movements from classes with high falls risk (**Self-reported Faller_m** and **Clinically At-Risk**) it made substantial errors.

**Figure 6:**
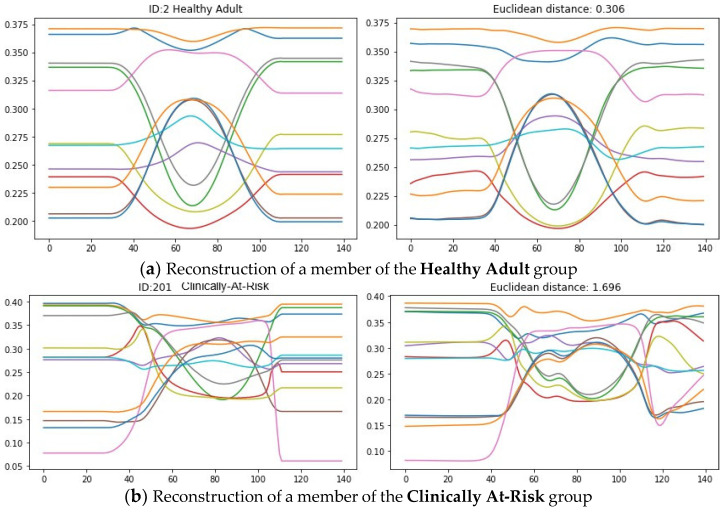
**Reconstruction of single rep:** This Figure shows the reconstruction of a single repetition carried out by (**a**) a member of the **Healthy Adult** group and (**b**) a member of the **Clinically At-Risk** group. Movement (**a**) was part of the validation set, movement (**b**) was part of the test set. The Autoencoder is able to recreate movement (**a**) with high faithfulness, and so the reconstruction error (expressed as Euclidean distance) is low. The autoencoder struggles to recreate movement (**b**) because it comes from a different distribution; the reconstruction error is 5.5 times larger for this movement, indicating that it lies far away from the training distribution. The time series shown here have been normalised to aid training. Hence, the *y*-axis shows normalised joint angles, and the *x*-axis shows frames. *Note, the first 30 and last 30 frames, of the reconstructed time series, are clipped before the distance is calculated; these frames were added as part of the padding process and do not represent the movement*.

The reconstruction error was quantified using Euclidean distance (ED) between the original signal and the output. Figure 6 shows input and reconstructed signals for unseen examples of the **Healthy Adult** and the **Clinically At-Risk** groups.

### 3.6. Scoring Falls Risk

The reconstruction error of individual repetitions was not enough to consistently identify individuals with an elevated falls risk, i.e., some repetitions of those in the at-risk groups can be close to normal, while others lay far away.

To address this issue, we propose a scoring system that utilises the variance between repetitions as a key indicator of falls risk. The score is calculated using Equation (2). The scoring system multiplies the reconstruction error value by the variance of the reconstruction error between repetitions. Including the variance reflects the fact that healthy individuals are more able to carry out the sit-to-stand movement consistently, showing low variance between repetitions, and those at risk of falls are less consistent, showing high variance between repetitions. Subtracting this term from 1 makes the maximum score of 1 achievable, and falls risk is placed on a scale of <1. Averaging the per-repetition scores gives the final score.

(2) $$\frac{1}{n}\sum 1-(ED\times \sigma_{ED})$$

where *ED* is the Euclidean distance between input and reconstruction, *σ_ED_* is the variance between *ED* over the total number of repetitions, and *n* is the number of repetitions from each recording.

## 4. Results

**Figure 7:**
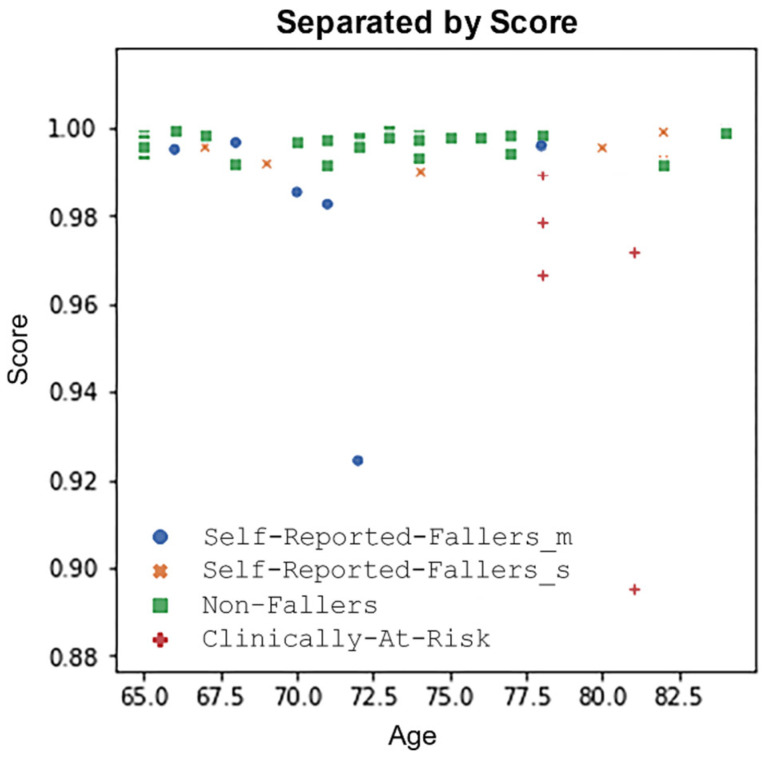
**Separation of individuals by impairment:** this graph represents the score derived from the autoencoder; the most at-risk are shown furthest away from the normal threshold, indicated by the blue line.

Applying the scoring system to the older members of the KINECAL dataset (all those over 65 years), a pattern emerged (Figure 7). The **Non-fallers** and **Self-Reported Fallers_s** scored close to 1, while the most at-risk groups (**Self-Reported Fallers_m** and **Clinically At-Risk**) showed lower scores. This points to the use of the scoring system to identify those at the highest risk of future falls. A similar overlap between those who report no falls and those who report only a single fall was noted in the work of Buatois et al. [32]. Multiple fallers are likely to fall again. On the other hand, single fallers are more likely to have just been unlucky in a fall and so have more in common with non-fallers.

### 4.1. Obtaining a Threshold Value

To discover a threshold that would identify those at elevated falls risk, we defined a binary classification task. We combined the groups together to create two classes, i.e., **At-risk** and **Not-At-risk**. The **Self-reported Faller_m** and the **Clinically At-Risk** were grouped and labelled as the **At-risk** class. Similarly, the **Non-Fallers** and **Self-reported Faller_s** were grouped and labelled as the **Not-At-risk** class. The rationale for these groupings can be seen both in Figure 7 and in the work of Buatois et al. [32].

**Figure 8:**
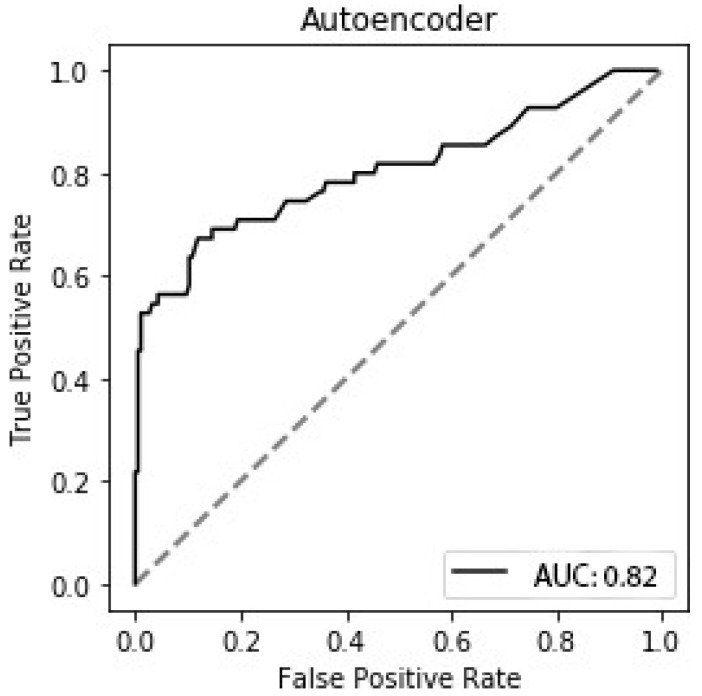
**ROC curve for the proposed model:** the ROC curve shows the trade-off between false positive rate and true positive rate for different threshold values. A threshold of 0.991 was found to give the best result. This threshold gave an average specificity of 0.88 and an average sensitivity of 0.68, shown in Table 1.

Using the range of the scores, shown in Figure 7 (0.88 to 1.00), as thresholds, we were able to create a receiver operator curve (ROC) (Figure 8). Using this ROC, we found that 0.991 was the threshold that gave the best balance between the true positive rate and the false positive rate and an AUC of 0.82. Hence, this is the threshold a clinician could use to identify individuals at elevated falls risk.

### 4.2. Classification of Those with Elevated Falls-Risk

**Figure 9:**
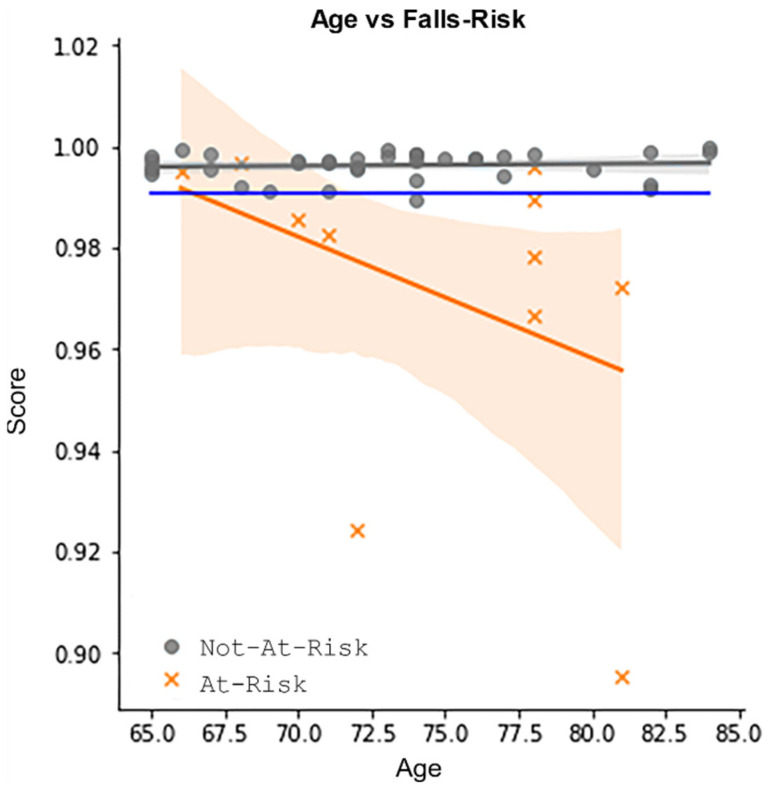
**Graph of age vs. falls risk:** this graph shows two distinct populations, **Not-At-Risk** (grey) and **At-Risk** (orange). The trend line for both is very different with age. The model separates the two classes based on whether the score lies above or below a threshold value, shown as a blue line.

By re-plotting the data using the two classes and marking the threshold value as a blue line, we obtain Figure 9. To obtain average model metrics, we carried out a 5-fold cross-validation. Using the same threshold value of 0.991, we obtained an average accuracy of 0.84, specificity of 0.88, and sensitivity of 0.68, shown in Table 1.

Using the proposed scoring system, we were able to separate those at risk of falling from those not at risk of falling, given that they are 65 years and older. Those in the **Not-At-Risk** class demonstrated a high score across an age range of 65–85. Conversely, the **At-Risk** class show a lower score on average, with the score decreasing with age.

## 5. Discussion

In this study, we propose a scoring system for the assessment of falls risk based on the output of an objective representational model (an LSTM autoencoder). It was trained to reconstruct a time series of joint angles as they change over time. As training data, we used joint angles derived from healthy adults carrying out the STS-5 test. During training, the autoencoder built a representational model of an idealised healthy adult carrying out 1 repetition of the STS-5 test. The reconstruction error from this model provides an indicator of how far away from the learned norm a particular participant’s movements are. We went on to develop a scoring system that takes into account not only information derived from the model but also the variance between repetitions of the sit-to-stand movement.

Figure 4 compares a single repetition of the STS-5 movement performed by a **Healthy Adult** and a **Clinically At-Risk** individual. The graph on the left (**Healthy Adult**) demonstrates smooth transitions in all joints, with hip joints (red lines) and knee joints (blue lines), following similar arcs. For the healthy adult, the joint angles are constrained by the body’s own systems and never reach their physical limits. The graph on the right (**Clinically At-Risk**) is much less well organised; there is not as much synchronicity in the hip and knee joints, and the joints experience a greater range of movement. The knee joints can be seen reaching their physical limits and then recoiling to recapture some needed degrees of freedom. This demonstrates discoordination and lack of control over movements for the at-risk individual. Discoordination and a reduction in muscular control are precursors to the development of frailty and are indicative of falls [33,34].

In our work, we use the information derived from both upper and lower body joints. A visual inspection of Figure 4 shows clear differences between a member of the Healthy Adult group and the Clinically At-Risk group. Given enough time and lots of cross-checking, one could create a set of rules that relate the change in angle through the motion to falls risk. Our research seeks to automate this process and provide a useful measure. By training the autoencoder to recreate the motions of healthy adults, it was necessary to build an internal model of healthy movements [35,36]. As well as clear differences between individuals, there was more variation between repetitions for those at risk of falling. We utilised this fact in the proposed scoring system by including a measure of variance between the repetitions.

A threshold was calculated for the proposed scoring system, below which individuals show elevated falls risk. However, this should not be seen as a cliff edge, as some individuals might be able to accommodate impairment better than others. This highlights the fact that systems, such as the one detailed here, should be an aid to health professionals and not a replacement for clinical judgement. That being said, using the threshold as a means of classification, we obtained an accuracy of 0.84, AUC of 0.82, specificity of 0.88 and sensitivity of 0.68. These are similar results to the recent paper from Zhang et al. [37]. However, they used a combination of gait metrics and personal information, such as age, sex, etc., whereas our method uses just the information derived from the movement. In addition, gate analysis needs a far bigger area in which to carry out the test than the sit-to-stand test. Hence, our use of just the sit-to-stand test makes it more appropriate for use in the limited space of a doctor’s office or clinic. A similar result was also obtained by [38], using data from a timed up and go (TUG) test, which includes a sit-to-stand phase. However, their method required the use of both Kinect and a tri-axial accelerometer to achieve the result we reported with just Kinect alone. Both of these studies used hand-crafted features, suggested by experts. In contrast, our method learns features automatically, directly from the data.

Figure 9 demonstrates how the proposed method can be used to identify those at risk of falls in an age range of 65 to 85. It also identifies participants who, despite being in the same age range, move like a healthy adult under the age of 65 and, as such, are at low risk of falls. This type of research, which differentiates between age-related measures and impairment-related measures, was a stated aim of the design of the KINECAL dataset.

The proposed method provides a score of how at risk an individual might be. In this study, we suggest a threshold under which someone is at risk of falls. However, theinclusion of a margin might prove useful in practical applications. Another advantage of this approach over a pure categorisation approach is that it gives a score on a continuous scale, which can be used not only to identify potential fallers but also to track improvements following interventions.

The limitations of this work are as follows:

- The model used in this study was trained on the KINECAL dataset, which, while the largest of its kind, is still small. Future work will endeavour to gather more examples of people at high falls risk. Future work should seek to validate these results in larger populations.
- The data used in this study were collected from volunteers in the local community. Inevitably, these people do not represent the most at-risk individuals. The model demonstrated that even in these groups, differences could be discovered. Future work will seek to validate this model using a wider range of participants.
- The single repetition extraction process relies on a final visual inspection to assure the quality of the data. Future work should consider fully automatic methods.

## 6. Conclusions and Future Work

This research represents an objective method for measuring fall risk. One can imagine a small device, derived from this research, that could sit in a doctor’s office and make the assessment of falls risk as commonplace as taking someone’s blood pressure. This type of device could provide the option of a referral to a falls clinic as a preventative measure before the first fall occurs.

Most state-of-the-art studies concentrate on gait analysis for the assessment of falls. However, gait analysis can be difficult to carry out in the confines of a small office. In this study, we have opted to use a sit-to-stand test that can be easily carried out in office-sized spaces.

Our proposed method not only provides a way of screening for future fallers but could also be used in the rehabilitation process to demonstrate how interventions are helping patients regain normal movement. As well as being a useful aid to health professionals, this type of feedback can help to ensure that the recommended exercises are carried out on a regular basis. If people can see progress, they are more likely to continue. Future work could consider if the inclusion of angular velocity and asymmetry between the left and right sides of the body could provide additional useful features.

## Figures and Tables

**Table 1:** **Performance metrics** A summary of 5-fold cross validation. The mean values are shown with standard deviation in brackets and a 95% confidence interval (CI).

	Accuracy	Specificity	Sensitivity
1	0.88	0.92	0.73
2	0.94	0.95	0.87
3	0.85	0.95	0.63
4	0.83	0.92	0.60
5	0.71	0.65	0.59
mean	0.84 (0.08) 95% CI ± 0.15	0.88 (0.11) 95% CI ± 0.22	0.68 (0.11) 95% CI ± 0.21

## Data Availability

The data used was generated and published separately as a publicly available dataset. This is available at: https://www.nature.com/articles/s41597-023-02375-w (accessed on 1 May 2024).
